# The Japanese Breast Cancer Society Clinical Practice Guidelines for Breast Cancer Screening and Diagnosis, 2018 Edition

**DOI:** 10.1007/s12282-019-01025-7

**Published:** 2019-11-16

**Authors:** Takayoshi Uematsu, Kazutaka Nakashima, Mari Kikuchi, Kazunori Kubota, Akihiko Suzuki, Shogo Nakano, Kouichi Hirokaga, Ken Yamaguchi, Shigehira Saji, Hiroji Iwata

**Affiliations:** 1 Division of Breast Imaging and Breast Intervention Radiology, Shizuoka Cancer Center, Shizuoka, Japan; 2 Department of General Surgery, Kawasaki Medical School General Medical Center, Okayama, Japan; 3 Department of Diagnostic Imaging, Cancer Institute Hospital, Tokyo, Japan; 4 Department of Radiology, Dokkyo Medical University Hospital, Tochigi, Japan; 5 Department of Breast and Endocrine Surgery, Tohoku Medical and Pharmaceutical University, Sendai, Japan; 6 Division of Breast and Endocrine Surgery, Department of Surgery, Aichi Medical University, Aichi, Japan; 7 Department of Breast Surgery, Hyogo Cancer Center, Hyogo, Japan; 8 Department of Radiology, Faculty of Medicine, Saga University, Saga, Japan; 9 Department of Medical Oncology, Fukushima Medical University, Fukushima, Japan; 10 Department of Breast Oncology, Aichi Cancer Center Hospital, Nagoya, Japan; 11 The Japanese Breast Cancer Society Clinical Practice Guidelines Breast Cancer Screening and Diagnosis Subcommittee, Tokyo, Japan; 12 The Japanese Breast Cancer Society Clinical Practice Guidelines Committee, Tokyo, Japan

**Keywords:** Japanese breast cancer society, Clinical practice guidelines, Breast cancer screening, Breast cancer diagnosis

## Abstract

This article updates readers as to what is new in the Japanese Breast Cancer Society Clinical Practice Guidelines for Breast Cancer Screening and Diagnosis, 2018 Edition. Breast cancer screening issues are covered, including matters of breast density and possible supplemental modalities, along with appropriate pre-operative/follow-up diagnostic breast imaging tests. Up-to-date clinical practice guidelines for breast cancer screening and diagnosis should help to provide patients and clinicians with not only evidence-based breast imaging options, but also accurate and balanced information about the benefits and harms of intervention, which ultimately enables shared decision making about imaging test plans.

## Introduction

The Japanese Breast Cancer Society Clinical Practice Guidelines for Breast Cancer Screening and Diagnosis, 2018 Edition provide consensus statement from groups (a guideline forming committee, a panel of experts, including breast cancer survivors, who rated statements according to the modified Delphi method; and, an evaluating committee) about their views on currently accepted approaches to breast cancer screening and diagnosis. The practice guidelines stress the importance of asking women who have no signs or symptoms of breast cancer and patients to share the decision-making process regarding all aspects of breast cancer screening and diagnosis. This article summarizes the practice guidelines, including eight clinical questions and one background question, supported by recommendations and evidence, along with the weight of consensus among the expert panelists and supporting references.

## Practice guidelines for breast cancer screening

### CQ1. Is handheld ultrasound recommended as an adjunct to population-based breast cancer screening mammography for women with dense breasts?

#### Recommendation

We advise against using handheld ultrasound as an adjunct to population-based breast cancer screening mammography for women with dense breasts. [Strength of Recommendation (SoR), 3; Strength of Evidence (SoE), moderate].

#### Justification

The objective of the Japan Strategic Anti-Cancer Randomized Trial (J-START), which is the world’s first large-scale randomized controlled trial of supplemental ultrasound population-based breast screening, is to investigate the efficacy of adding ultrasound to screening mammography in 72,998 healthy Japanese women in their 40s [1]. The results, therefore, can be generalized to women with dense breasts. Preliminary results from the J-START show that sensitivity is significantly higher in the intervention group than in the control group, whereas the specificity is significantly lower. Furthermore, more small invasive cancers were detected in the intervention group than in the control group and were more frequently at stages 0 and 1. Also, there was a reduction in interval cancers. However, breast cancer mortality rate reduction is the most important parameter that can be used to evaluate the efficacy of breast screening and no studies, including the J-START, have shown this. In addition, the low specificity of the intervention group reflects its high recall rate in the J-START. Consequently, the benefits [1, 2] of handheld ultrasound as an adjunct to population-based breast cancer screening mammography for women with dense breasts cannot outweigh the harm [3–6].

### CQ2. Is breast tomosynthesis recommended as an adjunct to population-based breast cancer screening mammography for women with dense breasts?

#### Recommendation

We advise against using breast tomosynthesis as an adjunct to population-based breast cancer screening mammography for women with dense breasts [SoR, 3; SoE, very weak].

#### Justification

Breast tomosynthesis is a pseudo-three-dimensional digital mammography imaging system that produces a series of 1-mm-slice images using multiple, very low-dose X-ray projections to reveal the inner architecture of the breast after eliminating interference from overlapping breast tissue. There is evidence that it improves cancer detection and decreases the recall rate in some studies [7–18]. However, no studies have shown that it reduces breast cancer mortality rates. Additionally, there has been no randomized controlled trial of supplemental breast tomosynthesis population-based breast screening. In the case of extremely dense breasts, some cancers remain difficult to identify by breast tomosynthesis [18]. A few studies have reported that breast tomosynthesis may be cost-effective for women with dense breasts [19, 20].

### CQ3. Is an automated whole-breast scanning sonography system recommended as an adjunct to population-based breast cancer screening mammography for women with dense breasts?

#### Recommendation

We advise against using automated whole-breast scanning sonography system as an adjunct to population-based breast cancer screening mammography for women with dense breasts [SoR, 3; SoE, very weak].

#### Justification

Automated whole-breast scanning sonography involves automated ultrasound technology for whole-breast imaging with safe, painless, radiation-free, and non-invasive three-dimensional ultrasound [21]. Evidence from four studies [22–25] suggested that adding automated whole-breast scanning sonography to mammography results in highly sensitive cancer detection capabilities with a high recall rate. However, no studies have shown that this reduces the breast cancer mortality rate. In addition, there has been no randomized controlled trial of supplemental automated whole-breast scanning sonography in population-based breast screening.

### CQ4. Is contrast-enhanced breast MRI screening as an adjunct to mammography recommended for Japanese BRCA1 and BRCA2 mutation carriers?

#### Recommendation

We advise using contrast-enhanced breast MRI screening as an adjunct to mammography for Japanese BRCA1 and BRCA2 mutation carriers [SoR, 2; SoE, weak].

#### Justification

The BRCA1 and BRCA2 mutation carriers have a 6–12 times higher lifetime risk of developing breast cancer compared to non-carriers [26]. In the USA and Europe, contrast-enhanced breast MRI screening as an adjunct to mammography for BRCA1 and BRCA2 mutation carriers has been established [27]. Intensive combined breast cancer screening with annual MRI and mammography has been shown to improve survival rates for BRCA2 mutation carriers [28]. Data from two prospective studies in which asymptomatic women who undertook a mammography alone or with MRI, compared with BRCA1 and BRCA2 mutation carriers with no intensive surveillance, showed that there were no differences in 10-year survival rates between the MRI + mammography and mammography-only groups, but survival was significantly higher in the MRI screened group compared to no intensive screening [29]. Consistent evidence from seven studies [30–36] demonstrated that sensitivity was significantly higher in the MRI screened group compared to the mammography-only group. The most important reason for this is that MRI is not affected by breast density and BRCA1 and BRCA2 mutation carriers are associated with a diagnosis of breast cancer at a young age, resulting in dense breasts. Additional screening sensitivity from mammography, over that obtained by MRI, is limited in BRCA1 mutation carriers, whereas mammography contributes to increased screening sensitivity in BRCA2 mutation carriers, especially those ≤ 40 years-of-age [37]. It may be reasonable to consider potential omission of mammography screening in BRCA1 mutation carriers. However, there are a few potential adverse events to consider before using contrast-enhanced breast MRI screening [38, 39]. In conclusion, the efficacy of risk-based breast cancer screening practices, such as MRI, for BRCA1 and BRCA2 mutation carriers shows promise in terms of increased cancer detection rates and decreased mortality.

## Practice guidelines for breast cancer diagnosis

### CQ5. Is breast elastography as an adjunct to B-mode ultrasound recommended in a diagnostic setting?

#### Recommendation

We advise using breast elastography as an adjunct to B-mode ultrasound recommended in a diagnostic setting [SoR, 2; SoE, weak].

#### Justification

Consistent evidence from 10 studies [40–49] demonstrated that addition of elastography to B-mode ultrasound can increase the negative predictive value of diagnostic breast ultrasound in women, while reducing the number of false-positive findings without missing cancers. Breast elastography is expected to reduce the number of unnecessary biopsies and contribute to an increase in the accuracy of diagnostic breast ultrasound.

### CQ6. Is contrast-enhanced breast MRI recommended in a diagnostic setting?

#### Recommendation

We advise using contrast-enhanced breast MRI in a diagnostic setting [SoR, 2; SoE, weak].

#### Justification

No evidence was found from 11 studies, including 1 randomized controlled trial [50–60], that the addition of MRI to conventional imaging and clinical examination has benefits in reducing the locoregional recurrence. Evidence from eight studies [61–68] showed that MRI could provide very valuable information for pre-operative planning and single-stage resection in breast cancer. One meta-analysis study showed that MR imaging of the breast provides high sensitivity (90%) and low specificity (72%) in the evaluation of breast lesions [69]. The prevalence of malignancy among MRI-detected lesions is not negligible with reported rates ranging between 52% and 66% [70–73]. Three studies [52, 55, 57] reported that there was no statistically significant effect of the use of pre-operative MRI on rates of contralateral recurrence or disease-free survival. However, another three studies [51, 58, 74] demonstrated that there was a statistically significant effect of the use of pre-operative MRI on rates of contralateral recurrence or disease-free survival. As a special circumstance in Japan, Japan universal health insurance covers contrast-enhanced breast MRI in a diagnostic setting and MRI is, therefore, widely used in this country. In light of the above, contrast-enhanced breast MRI can be recommended in a diagnostic setting under the quality assurance and quality control recommendations of Japan.

### CQ7. Is intensive staging imaging including CT, PET, and PET-CT recommended to detect asymptomatic distant metastases for women with stage 1 and 2 breast cancers?

#### Recommendation

We advise against using intensive staging imaging, including CT, PET, and PET-CT, to detect asymptomatic distant metastases for women with stage 1 and 2 breast cancers [SoR, 3; SoE, very weak].

#### Justification

A low prevalence of distant metastases was seen in stage 1 and 2 breast cancers and the prognostic effect of using intensive staging imaging was far from clear [75–82]. The benefit of using intensive staging imaging to avoid unnecessary surgery cannot outweigh the harm, resulting in false-positive findings, further costs, and further radiation exposure. In the future, intensive staging imaging after a new diagnosis of stage 1 and 2 breast cancers may be necessary depending on the subtype classification to avoid over- and under-treatment [83].

### CQ8. Is intensive imaging recommended for surveillance and follow-up of patients with stage 1 and 2 breast cancers?

#### Recommendation

We advise against using intensive imaging for the surveillance and follow-up of patients with stage 1 and 2 breast cancers [SoR, 3–4; SoE, weak].

#### Justification

The American Society of Clinical Oncology recommends against performing surveillance testing (biomarkers) or imaging (PET, CT, and radionuclide bone scans) for asymptomatic individuals who have been treated for breast cancer with curative intent [84]. The European School of Oncology states that early detection of metastatic lesions is not a valuable end point in itself and if intensive surveillance is to be recommended, it must be associated with some direct patient benefit [85]. No evidence was found from 3 studies [86–88] that intensive imaging for surveillance and follow-up of patients with stage 1 and 2 breast cancers can improve survival or influence health-related quality of life. A small study reported that the percentage of first relapse cases detected by imaging or tumor markers for stage 1, 2A, and 2B was 4.7, 5.1, and 11.8, respectively [89]. However, considering the progress made in the treatment of metastatic disease and the rapid evolution of targeted therapy that requires customization of the treatment strategy according to molecular characteristics of the disease, patients could derive real benefits from the early detection of disease recurrence [90]. A new study in Japan is being conducted to determine the superiority of intensive follow-up over standard follow-up in terms of overall survival for patients with high-risk breast cancers [91]. Using intensive imaging leads to increased costs and radiation exposure [86, 92, 93].

## Practice guidelines for breast cancer diagnosis

### BQ1. Is image-guided breast biopsy recommended to use in breast cancer diagnosis instead of open surgical biopsy?

#### Statement

We recommend that image-guided breast biopsy must generally be used for breast cancer diagnosis instead of open surgical biopsy.

#### Justification

Consistent evidence from six studies [94–99] demonstrated that image-guided breast biopsy is almost as accurate as open surgical biopsy with lower complication rates.
