# Correction to: The Japanese Breast Cancer Society Clinical Practice Guidelines for Breast Cancer Screening and Diagnosis, 2018 Edition

**DOI:** 10.1007/s12282-021-01251-y

**Published:** 2021-05-19

**Authors:** Takayoshi Uematsu, Kazutaka Nakashima, Mari Kikuchi, Kazunori Kubota, Akihiko Suzuki, Shogo Nakano, Kouichi Hirokaga, Ken Yamaguchi, Shigehira Saji, Hiroji Iwata

**Affiliations:** 1 Division of Breast Imaging and Breast Intervention Radiology, Shizuoka Cancer Center, Shizuoka, Japan; 2 Department of General Surgery, Kawasaki Medical School General Medical Center, Okayama, Japan; 3 Department of Diagnostic Imaging, Cancer Institute Hospital, Tokyo, Japan; 4 Department of Radiology, Dokkyo Medical University Hospital, Tochigi, Japan; 5 Department of Breast and Endocrine Surgery, Tohoku Medical and Pharmaceutical University, Sendai, Japan; 6 Division of Breast and Endocrine Surgery, Department of Surgery, Aichi Medical University, Aichi, Japan; 7 Department of Breast Surgery, Hyogo Cancer Center, Hyogo, Japan; 8 Department of Radiology, Faculty of Medicine, Saga University, Saga, Japan; 9 Department of Medical Oncology, Fukushima Medical University, Fukushima, Japan; 10 Department of Breast Oncology, Aichi Cancer Center Hospital, Nagoya, Japan; 11 The Japanese Breast Cancer Society Clinical Practice Guidelines Breast Cancer Screening and Diagnosis Subcommittee, Tokyo, Japan; 12 The Japanese Breast Cancer Society Clinical Practice Guidelines Committee, Tokyo, Japan

## Correction to: Breast Cancer (2020) 27:17–24 10.1007/s12282-019-01025-7

The article “The Japanese Breast Cancer Society Clinical Practice Guidelines for Breast Cancer Screening and Diagnosis, 2018 Edition”, written by Takayoshi Uematsu, Kazutaka Nakashima, Mari Kikuchi, Kazunori Kubota, Akihiko Suzuki, Shogo Nakano, Kouichi Hirokaga, Ken Yamaguchi, Shigehira Saji, Hiroji Iwata, was originally published electronically on the publisher’s internet portal on 16 November 2019 without open access. After publication in volume 27, issue 1, page 17–24, the author decided to opt for Open Choice and to make the article an Open Access publication. Therefore, the copyright of the article has been changed to © The Author(s) and the article is forthwith distributed under the terms of the Creative Commons Attribution 4.0 International License, which permits use, sharing, adaptation, distribution and reproduction in any medium or format, as long as you give appropriate credit to the original author(s) and the source, provide a link to the Creative Commons licence, and indicate if changes were made.

The images or other third party material in this article are included in the article’s Creative Commons licence, unless indicated otherwise in a credit line to the material. If material is not included in the article’s Creative Commons licence and your intended use is not permitted by statutory regulation or exceeds the permitted use, you will need to obtain permission directly from the copyright holder.

To view a copy of this licence, visit http://creativecommons.org/licenses/by/4.0/

The original article has been updated.

